# Analysis of word number and content in discourse of patients with mild to moderate Alzheimer's disease

**DOI:** 10.1590/S1980-57642014DN83000010

**Published:** 2014

**Authors:** Juliana Onofre de Lira, Thaís Soares Cianciarullo Minett, Paulo Henrique Ferreira Bertolucci, Karin Zazo Ortiz

**Affiliations:** 1 PhD. Speech Therapist, Department of Speech Therapy, Federal University of São Paulo, SP, Brazil.; 2 M.D, PhD. Senior Researcher. University of Cambridge, Cambridge, UK.; 3 M.D, PhD. Professor, Sector of Behavioral Neurology, Department of Neurology and Neurosurgery, Federal University of São Paulo, SP, Brazil.; 4 Speech Therapist, PhD. Professor, Department of Speech Therapy, Federal University of São Paulo, São Paulo, SP, Brazil.

**Keywords:** Alzheimer's disease, dementia, language

## Abstract

Alzheimer's disease (AD) is characterized by impairments in memory and other cognitive functions such as language, which can be affected in all aspects including discourse. A picture description task is considered an effective way of obtaining a discourse sample whose key feature is the ability to retrieve appropriate lexical items. There is no consensus on findings showing that performance in content processing of spoken discourse deteriorates from the mildest phase of AD.

**Objective:**

To compare the quantity and quality of discourse among patients with mild to moderate AD and controls.

**Methods:**

A cross-sectional study was designed. Subjects aged 50 years and older of both sexes, with one year or more of education, were divided into three groups: control (CG), mild AD (ADG1) and moderate AD (ADG2). Participants were asked to describe the "cookie theft" picture. The total number of complete words spoken and information units (IU) were included in the analysis.

**Results:**

There was no significant difference among groups in terms of age, schooling and sex. For number of words spoken, the CG performed significantly better than both the ADG 1 and ADG2, but no difference between the two latter groups was found. CG produced almost twice as many information units as the ADG1 and more than double that of the ADG2. Moreover, ADG2 patients had worse performance on IUs compared to the ADG1.

**Conclusion:**

Decreased performance in quantity and content of discourse was evident in patients with AD from the mildest phase, but only content (IU) continued to worsen with disease progression.

## Abstract (pt)

Doença de Alzheimer (DA) é caracterizada por prejuízo na memória e em outras funções cognitivas, como a linguagem, que pode ser afetada em todos os aspectos, incluindo o discurso. Tarefa de descrição de figura é considerada uma forma eficaz de obter amostra de discurso cuja característica fundamental é a capacidade de recuperar itens lexicais adequados. Não há consenso nos achados sobre deterioração no desempenho do conteúdo do discurso na fase leve da DA.

**Objetivo:**

Comparar a quantidade e a qualidade do conteúdo do discurso em pacientes com DA leve, moderada e controles.

**Métodos:**

Foi feito um estudo transversal cuja amostra foi composta por indivíduos a partir de 50 anos, de ambos os sexos, com um ou mais anos de escolaridade. Foram divididos em três grupos: controle (GC), DA leve (ADG1) e DA moderada (ADG2) e a eles foi solicitado descrever a "prancha do roubo dos biscoitos". Considerados na análise o número total de palavras completas faladas e o de unidades de informação (UI).

**Resultados:**

Não houve diferença significativa entre os grupos. Para o número de palavras, o CG apresentou desempenho significativamente melhor que AD1 e AD2, não houve diferença entre os dois últimos grupos. O GC produziu quase o dobro de unidades de informação em relação ao ADG1 e mais que o dobro do que ADG2. Além disso, o ADG2 apresentou pior desempenho nas UI em comparação ao ADG1.

**Conclusão:**

Foi observado um evidente prejuízo no desempenho quanto à quantidade e ao conteúdo do discurso em pacientes com DA a partir da fase leve. Entretanto apenas o conteúdo continuou a se agravar com a progressão da doença.

## INTRODUCTION

Dementia due to Alzheimer's disease (AD) is a complex progressive degenerative brain disorder affecting multiple cerebral functions. It is considered the most common cause of dementia and affects between 5 and 8% of elderly over 60 years of age.^1^ According to the criteria of the National Institute of Neurological Communicative Disorders and Stroke/Alzheimer's Disease and Related Disorders Association (NINCDS/ADRDA) for the diagnosis of probable AD, besides memory changes, the patient must necessarily display impairment in at least one other cognitive function, which may be language.^2^

Language disturbances have been cited as one of the most important cognitive deficits among patients with AD.^3^ Thus, language assessment is included in several neuropsychological batteries. These batteries, however, tend not to cover the broader aspects of language, particularly the functional linguistic aspect.^4^ While analyses of language skills based on tasks such as picture and object naming in isolation from everyday discourse provide limited information on lexical access, analysis of language in the context of connected speech is more ecologically valid and provides more complete information about the integration of cognitive–linguistic abilities, a particularly relevant aspect in dementia. According to Giles et al.,^5^ discourse ability entails a complex interaction of linguistic, communicative and other cognitive processes where a picture description task is considered the most effective way of obtaining a suitable discourse sample that can be standardized across many subjects. There are at least four benefits of using this instrument:

(i) it provides a clear pictorial focus, thus reducing ambiguity about the subject matter;
(ii) it reduces the demand on memory because the stimulus remains available to the subject at the time of evaluation;
(iii) it minimizes confounders in analysis due to the controlled nature of the speech content; and
(iv) when used to reevaluate, it monitors progression.^6,7^

One of the most used tools to elicit discourse is the Cookie Theft picture (CTP) from the Boston Diagnostic Aphasia Examination.^8^ The picture depicts a familiar domestic scene with basic key vocabulary learned in childhood with distinct characters, time, and place contrasts.^5^ Although it was originally devised for use with aphasic patients, the CTP has proved its usefulness in clinical research with various groups of diseases, including AD, and in several different languages.

According to Mackenzie,^6^ a major element of the picture description task is the ability to retrieve appropriate lexical items. One of the most known variables is information units which are "words that are intelligible in context and accurately convey information relevant to eliciting stimulus".^9^ Through the picture description task, discourse can also be quantified, usually by frequency of syllables or total number of complete words.

Several studies were conducted considering content and quantitative analysis in AD patients compared to normal controls, as well as for other diseases. Nicholas et al.^10^ compared AD patients with normal controls (NC) and against patients with Wernicke´s and anomic aphasia. The authors found that NC produced significantly more content elements than patients with AD and aphasia while detecting no differences among groups regarding number of total words. Croisile et al.^11^ measured the number of words and amount of information spoken by moderate AD patients compared to NC and found that controls produced more words and higher information content than AD patients. Kavé and Levy^12^ compared the description of the Cookie Theft Picture and found that participants with mild AD provided fewer information units than control participants. The authors concluded that although subjects with AD do not differ from controls when an information category includes a limited number of possible units, as the maximum number of information units increases, the differences between the groups also increase.

Other studies have considered the stages of AD in the analysis of discourse. Hier et al.^13^ found that patients with dementia (AD and vascular dementia) spoke fewer total words than healthy controls, but comparison among the AD patients showed no worsening of performance with increasing severity; in terms of content, AD patients produced fewer relevant observations on the CTP than controls, further decreasing with disease progression. Giles et al.^5^ found significant differences between controls and individuals with AD regarding information content spoken on the CTP, evident from the mildest phase of the disease. The authors also measured the number of syllables and noted that only moderate AD patients spoke a lower total number of words than healthy controls. Bschor et al.^14^ also found that patients with AD provided less relevant information from the mildest stage on, although the number of words produced remained unchanged. Groves-Wright et al.,^15^ using description of the CTP, observed that moderate AD subjects achieved significantly lower global scores than mild AD subjects and controls.

Taken together, the results of the studies cited above show that, in terms of quantitative and qualitative aspects, there is no consensus on the findings.

Therefore, the objective of this research was to compare quantitative and qualitative aspects of discourse among AD patients at mild and moderate stages and in healthy adult controls.

## METHODS

This was a cross-sectional study carried out at the outpatient clinic in the Behavioural Neurology Division of the São Paulo Federal University. The study was approved by the local Research Ethics Committee. After receiving full information about the study, written informed consent was obtained from all enrolled subjects or their carers in case of mental incapacity.

**Subjects.** The sample consisted of control (CG) and AD (ADG) groups. The general inclusion criteria were: age ≥50 years; schooling ≥1 year; no history of alcoholism or drug use; no use of psychotropic medications, except atypical neuroleptics; and absence of visual or uncorrected auditory impairments that might affect the outcome of the cognitive tests.

To evaluate general cognition and avoid the inclusion of patients with obvious dementia, the Mini-Mental State Examination (MMSE) was used as a screening tool. We adopted a Portuguese-translated version, with cut-off scores adjusted for subjects' educational level:^16^ illiterate - 20; elementary (1 to 4 years of education) - 25; 5 to 8 years of education - 26.5; 9 to 11 years of education - 28; and high level (more than 12 years) – 29 points.

The CG consisted of healthy elderly volunteers without neurological or psychiatric disturbances. Controls were all functionally independent, confirmed by scoring zero on the Pfeffer Questionnaire^17^ and cognitively preserved according to education-adjusted scores on the MMSE.

The ADG group consisted of patients diagnosed as having probable AD based on the criteria of the NINCDSARDRA.^18^ Only those individuals with an MMSE score higher than 12 and undergoing treatment for AD with a therapeutic dose of acetyl cholinesterase inhibitors (Donepezil ≥5 mg, Rivastigmine ≥9 mg or Galantamine ≥8 mg) were selected. The AD group was subdivided into two subgroups, according to MMSE score:

ADG1 (mild AD): comprising patients whose scores on the MMSE were from two to four standard deviations according to the local norms for age and education proposed by Brucki et al.^16^

ADG2 (moderate AD): comprising those individuals whose scores on the MMSE were from four to eight standard deviations according to the local norms for age and education proposed by Brucki et al.^16^

**Procedure.** Subjects were asked to describe the "Cookie Theft" picture – CTP.^19^ The instruction given was: "Could you tell me everything you are seeing in this figure." The test began the moment the subject started to describe the scene and finished when the individual indicated that there was nothing more to be said. Interruptions by the researcher were avoided and encouragement was provided as needed. The discourse was taped and later transcribed for analysis, taking into account all sample uttered by the subject, including repetitions, hesitations and help solicitation.

The total quantity of complete words was considered for the analysis, as was the total number of information units (IU). The IUs represent the amount of information provided by the subject. One point was attributed for each IU spoken by the subject, even when this was mentioned two or more times.

The 25 IUs proposed by Kave and Levy^12^ were used. These units were divided into four categories: subjects (boy, girl, mother), places (kitchen, exterior seen through the window), objects (cabinet, cookies, counter, curtain, dishes on the counter, faucet, floor, jar, plate, sink, stool, water, window) and actions (boy taking the cookie, boy or stool falling, woman drying or washing dishes/plate, water overflowing or spilling, the girl asking for a cookie, woman unconcerned by the overflowing, woman indifferent to the children). When synonymous expressions were used, these were accepted (e.g. ‘mammy' and ‘mother').

**Statistical analysis.** One-way ANOVA or Kruskal-Wallis tests were used to compare the total number of words and information units in the three groups: CG, ADG1 and ADG2. As both tests disclosed similar results, only the parametric results are shown. *Post-hoc* analysis was performed using Tukey's test.

Multiple linear regression analyses were used to investigate the individual relationships between independent and dependent variables, where study group, age, sex and years of education were independent variables, and number of spoken words and information units were the dependent variables. Among the study groups, ADG1 was chosen as a reference. The assumptions of these analyses were verified.

The Chi-square (χ^2^) test (without correction of Yates) was used to compare the categorical variables: sex and each information unit.

A p-value of less than 0.05 was considered to indicate statistical significance; all tests were two-tailed. Ninety-five percent of the confidence interval (CI) was calculated for the regression coefficients. All statistical analyses were carried out using the statistical software SPSS (Statistical Package for the Social Science) 13.5.1for Windows.

## RESULTS

For this study, 63 individuals were screened: 26 healthy elderly volunteers and 37 AD patients. Six subjects were excluded from the control group and eleven from the ADG because their MMSE scores did not fulfill the inclusion criteria for this study. Therefore, 26 patients with AD and 20 controls participated in the study.

The CG was compared with two groups of AD patients for performance on the MMSE: 15 subjects in the ADG 1 and 11 subjects in the ADG2.

**Sociodemographic conditions.** As shown in Table 1, no statistically significant difference was found among ADG1, ADG2 and CG, regarding age and education. As outlined, ADG2 patients had significantly lower scores on the MMSE than ADG1 patients, who in turn had lower scores than controls.

**Table 1:** Comparison among groups according to age, education, MMSE, total words and IU.

Groups Variables	CG Mean (SD)	ADG1 Mean (SD)	ADG2 Mean (SD)	F	p
Age	71.1(5.2)	68.3(9.8)	75.7(7.8)	3.0	0.060
Education	6.2(4.3)	7.3(5.3)	4.0(2.5)	1.8	0.171
MMSE*	27.6(1.6)	23.7(2.3)	15.7(2.4)	115.6	£0.001
Total words	99.0(50.6)	39.3(17.1)	4.0(2.5)	16.6	£0.001
IU	13.7(3.8)	8.0(2.8)	4.5(3.6)	25.5	£0.001

* MMSE: Mini-mental state examination.

There were no significant differences for sex among the study groups (CG – 51.6%, ADG1 – 25.8% and ADG2 – 22.6% of women; χ^2^(2)=2.86; p=0.232).

**Number of words and Information units.** Significant differences were found between the study groups regarding the number of words and IUs (Table 1). According to the *post-hoc* analysis (Tukey test), the CG performed significantly better than the ADG 1 (CI=29.7 to 89.6; p<0.001*) and the ADG2 (CI=32.3 to 100.3; p<0.001*) on number of words, but no significant differences were observed between the two AD groups (CI=-29.1 to 42.4; p=0.895). The CG produced almost twice the number of information units than ADG1 (CI=2.7 to 8.5; p<0.001*) and more than double that of ADG2 (CI=5.9 to 12.3; p<0.001*). Moreover, patients with ADG2 performed worse than ADG1 for total number of IUs (CI=0.15 to 6.9; p=0.039*).

Linear regression analysis was carried out to verify whether the groups and discourse performance relationships held after controlling the analysis for age, sex and education (Table 2).

**Table 2:** Results of linear regression analysis for study groups and discourse performance controlling for sex, age and education.

		β	SE	t	95% CI(b)			p
**Number of words**	CG	61.02	11.91	5.12	36.93	to	85.12	<0.001*
	ADG1 (ref)							
	ADG2	–1.34	14.77	–0.09	–31.23	to	28.53	0.928
	Age	1.01	0.76	1.33	–0.52	to	2.55	0.191
	Education	3.72	1.29	2.88	1.11	to	6.33	0.006*
	Male	0.39	12.86	0.30	–25.62	to	26.40	0.976
**Number of information units**	CG	5.50	1.08	5.05	3.30	to	7.70	<0.001*
	ADG1 (ref)							
	ADG2	–3.36	1.33	–2.52	–6.05	to	–0.67	0.016*
	Age	0.16	0.06	2.36	0.02	to	0.30	0.023*
	Education	0.42	0.11	3.60	0.18	to	0.66	0.001*
	Sex	0.60	1.13	0.53	–1.68	to	2.88	0.596

The presence of AD was associated with a lower number of words and IUs, independently of sex, age and education. Compared to mild AD patients (AD1), healthy individuals produced approximately 61 words and 6 IUs more, whereas moderate AD patients (AD2) produced 1 fewer word and 3 fewer IUs.

We also found a significant influence of schooling on the dependent variables, with an increase of approximately 4 words and 0.5 IU for each additional year of education. We also observed that age only interfered with number of information units.

## DISCUSSION

The main finding of this study was a decline in both the quantity and content of speech in patients with AD in relation to NC. However, a difference between mild and moderate stages was seen only for the qualitative aspect.

AD patients spoke fewer words than healthy individuals, but there was no difference in this aspect between mild and moderate AD groups. This same outcome was observed by Hier et al.^13^ utilizing the CTP but this finding is not a consensus. When comparing AD to controls, other studies, such as those carried out by Nicholas et al.,^10^ Bschor et al.^14^ and Feyereisen et al.,^20^ showed that patients with AD had similar performance to healthy subjects in terms of words spoken. However, differences in the methodologies used may explain this discrepancy. In the three studies cited, the AD group had more years of education than our group while Bschor et al.^14^ ranked the AD groups according to a functional scale (Global Deterioration Scale) and not the MMSE. Other researchers reporting worse performance in individuals with AD on number of words also considered the severity of AD in their analysis. In the study by Giles, Paterson and Hodges,^5^ patients with AD were divided into three groups and only the most severe produced fewer words than healthy subjects. Carlomagno et al.^21^ ranked their sample based on a different functional scale, the Clinical Dementia Rating, and found that subjects with moderate AD spoke fewer words than those with mild AD, who spoke fewer than control subjects. In the present study, the groups of patients with mild and moderate AD had similar performances. Thus, another possible explanation for the disparity observed between our study and others could be due to differences in stratification procedures. Therefore, we conclude that there is a deficit in discourse fluency in AD subjects compared with healthy individuals. Nonetheless, this alteration does not worsen with disease progression. Hier et al.,^13^ despite having found lower frequency in the AD group for total words, considered that the measure which best differentiated the two groups was number of semantic units. In our sample, AD patients produced fewer content elements than healthy individuals. This finding corroborates reports in the literature.^5,10-15,22^ Thus, we suggest a deficit in conceptual production of information in AD^21^ where the literature proposes some causes for this phenomenon. One of the most common reasons cited is the existence of impaired lexico-semantic processing caused by a deterioration in the related storage.^5,22-24^ Other authors, on the other hand, propose that an inability to access the semantic storage may occur.^25,26^ There is also a third hypothesis which proposes a failure in visual stimulus recognition or in access to the semantic system due to visual processing.^27,28^ Besides the semantic system, Glosser and Deser^29^ and Almor et al.^30^ affirm that degraded representations of verbal discourse depend on attention and executive control. Perry and Hodges^31^ stated that deficit in discourse information fits with the evidence that from initial stages of AD, attentional executive function is impaired. Indeed, we believe that all these hypothesis can plausibly explain the information deficit in discourse production.

Although performance for number of words was similar in both AD groups, when we observed the spoken content, this was seen to worsen with progression of the disease. This discrepancy between content and fluency in mild and moderate AD patients may be explained by the fact that quantity of discourse may have been maintained through the use of redundant words or those unrelated to the content, which Nicholas et al.^10^ have called "noninformative speech". This finding appears to be frequent in studies utilizing the CTP.^5,10,12-14,32^

We also found that education influenced number of words as well as information units. Mackenzie^6^ analyzed picture-supported discourse with the CTP in healthy individuals aged from 40 to 88 years and found that the less educated participants produced shorter and incomplete descriptions. In our sample, education significantly influenced the discourse in the healthy controls and AD participants.

Our study should be viewed in the light of some limitations. One such limitation is that the number of patients and controls was relatively small. This fact might have affected the results obtained, mainly the similarity of the AD groups on performance with information units.

Another limitation is that the mean education of our sample was lower than that of studies designed abroad. Despite the difference in this characteristic, it is important to consider this population of less educated aged individuals living in low and middle income countries (LAMIC) because, according to Ferri et al.,^33^ they are set to account for the majority of patients with dementia in the world.

Since patients with AD who are more educated perform better on cognitive tests than less educated patients, we were especially careful with our inclusion criteria and the process of stratification of AD subjects, by considering not only raw MMSE scores, but also their adequacy with the local education norms.

This study was relevant in confirming the underperformance of patients with AD compared to healthy subjects as well as the evolution of the discursive aspects in the disease. In addition, the task used is an important tool for investigating functional communication, with minimum memory requirements. Its analysis can be controlled and performance compared with patients with the same disease in other countries and various sociodemographic characteristics or even with repeat evaluations involving the same patient. Thus, the CTP has proved its usefulness in clinical research in AD.^5,6^

Discourse production (and comprehension) remains an important field for furthering knowledge on the dissolution of language in AD and a potential instrument for functional screening tools and therapeutic techniques to improve the lives of people living with dementia.
